# Microstructure and Antiwear Property of Laser Cladding Ni–Co Duplex Coating on Copper

**DOI:** 10.3390/ma9080634

**Published:** 2016-07-28

**Authors:** Yiyong Wang, Zhipeng Liang, Junwei Zhang, Zhe Ning, Hui Jin

**Affiliations:** College of Material and Metallurgy, University of Science and Technology Liaoning, Anshan 114051, China; wangyiyongfly@163.com (Y.W.); wangyiyong@ustl.edu.cn (Z.L.); lnkdzjw@163.com (J.Z.); classal@126.com (Z.N.)

**Keywords:** laser cladding, duplex coatings, metallurgical bonding, microhardness, wear resistance

## Abstract

Ni–Co duplex coatings were cladded onto Cu to improve the antiwear properties of Cu products. Prior to laser cladding, n-Al_2_O_3_/Ni layers were introduced as interlayers between laser cladding coatings and Cu substrates to improve the laser absorptivity of these substrates and ensure defect-free laser cladding coatings. The structure and morphology of the coatings were characterized by scanning electron microscopy and optical microscopy, and the phases of the coatings were analyzed by X-ray diffraction. Their hardness was measured using a microhardness tester. Experimental results showed that defect-free composite coatings were obtained and that the coatings were metallurgically bonded to the substrates. The surface of the Ni–Co duplex coatings comprised a Co-based solid solution, Cr_7_C_3_, (Fe,Ni)_23_C_6_, and other strengthening phases. The microhardness and wear resistance of the duplex coatings were significantly improved compared with the Cu substrates. The average microhardness of the cladded coatings was 845.6 HV, which was approximately 8.2 times greater than that of the Cu substrates (102.6 HV). The volume loss of the Cu substrates was approximately 7.5 times greater than that of the Ni–Co duplex coatings after 60 min of sliding wear testing. The high hardness of and lack of defects in the Ni–Co duplex coatings reduced the plastic deformation and adhesive wear of the Cu substrates, resulting in improved wear properties.

## 1. Introduction

Cu-based alloys are well known to be highly useful in the metallurgical and electrical industries because of their high thermal and electrical conductivities. For example, the excellent thermal conductivity of Cu–Cr–Zr alloy makes it a popular material for continuous casting molds. However, low slide wear resistance of Cu materials under severe conditions, especially under high temperatures, limits their application as crystallizers in continuous casting. Surface coatings provide a possible solution to the wear resistance problem of Cu materials and potentially extend the service life of Cu-containing components, thus reducing their total cost. Therefore, researchers’ interest in coating technologies involving electrodeposition, plasma spraying, and infiltration has been increasing. Although such methods can improve the wear resistance of Cu materials to a certain degree, their applications are limited because they rely on mechanical bonding between a coating and substrate. 

Laser surface cladding (LSC) on metal substrates is a nonequilibrium process with high cooling rates (103–108 K/s) that results in the formation of metastable phases by exceeding the solid-solubility limit beyond the equilibrium phase diagram through excellent metallurgical bonding to substrates [1,2,3,4]. Over the past few years, LSC has been demonstrated to represent a novel coating technology for improving the wear resistance of Cu substrates; thus far, Ni-, Co-, and Mo-based coatings have been reported [5,6,7]. Zhang et al. laser cladded Ni-based alloy onto pure Cu substrates with an average coating hardness of 360 HV [8]. Yan et al. laser cladded Co-based alloy/TiC/CaF_2_ self-lubricating composite coatings onto Cu alloys; resulting cladded alloys exhibited good friction-reducing and antiwear abilities at temperatures as high as 400 °C [9]. 

Although LSC technology has been used for continuous casting molds, obtaining large-surface-area, defect-free coatings on the surface of Cu substrates is difficult because of a high laser reflectivity of Cu materials and a low wettability of these materials on many metals. Laser cladding in conjunction with the introduction of an intermediate layer is a feasible approach for solving the absorption and wettability problems of Cu substrates. Ni has been reported to be more compatible with Cu substrates, and Ni-based alloys exhibit good laser absorption properties [10,11,12,13,14,15,16,17]. Therefore, in this study, we laser cladded Co-based alloys onto Cu substrates plated with n-Al_2_O_3_/Ni coatings as intermediate layers to improve the laser absorption characteristics of these substrates and overcome the incompatibility between Co-based coatings and Cu substrates. The Ni–Co duplex coatings exhibit good mechanical strength and surface hardness, excellent chemical durability, and high antiwear performance in high-temperature, corrosive environments.

## 2. Materials and Methods 

### 2.1. Materials

Surfaces of polished Cu plates (50 mm × 50 mm × 10 mm) cut from continuous casting molds were used as substrates; the composition of the plates was Cu–0.85Cr–0.25Zr (wt %). The samples were first degreased and activated in 10 wt % HCl solution and then rinsed with distilled water. Ni/n–Al_2_O_3_ coatings were then pre-electrodeposited onto the Cu substrates. Nanoparticulate Al_2_O_3_ with an average particle size of 20 nm and purity greater than 99.95% was used in the coatings. Electrodeposition parameters are as follows: solution temperature 30 °C, current density 3 A·dm^−2^, and deposition time 60 min. All reagents were of the AR grade. The composition of the plating solution is shown in Table 1.

**Figure 1:**
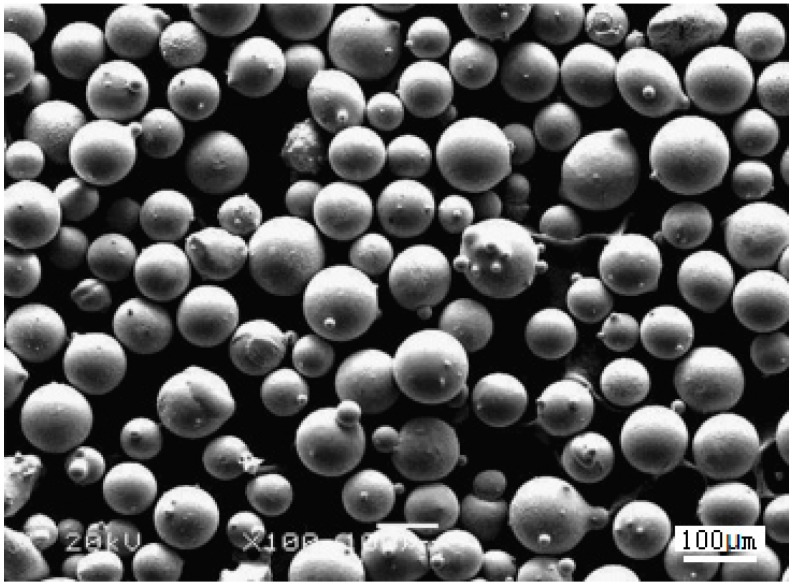
Micromorphology of the Co-based alloy powder used in the experiments.

Before laser cladding, the electroplated coatings were pre-heated. Thereafter, Co-based powders were laser cladded onto the Cu substrates using Ni/n–Al_2_O_3_ as an interlayer. Co-based alloy powder with a size distribution of 45–100 μm was used as a cladding material during laser cladding. The composition and micromorphology of the Co-based alloy powder are presented in Table 2 and Figure 1, respectively. Laser cladding experiments were conducted using a 6-kW transverse-flow CO_2_ laser (HGL-6000, HGTECH, Wuhan, China). A Gaussian pulse was used to obtain a stable circular laser spot. To obtain smooth, large-area LSC coatings, the cladding parameters were optimized as follows: Laser power 3.5 kW, laser beam diameter 4 mm, scanning rate 180 mm/min, and overlap ratio 40%.

### 2.2. Methods

After laser cladding, cross-sectional samples were prepared, polished, and etched in a solution of FeCl_3_ and Fe(NO_3_)_3_. The microstructure of the coatings was characterized by optical microscopy (Olympus GX71, Tokyo, Japan) and scanning election microscopy (SEM, JEOL JSM-840, Tokyo, Japan). SEM observations were conducted under an accelerating voltage of 15.0 kV using secondary electrons. The composition along the depth of the Cu substrates, from the Ni/n–Al_2_O_3_ coating to Co-based coating (Cu–Ni–Co), was measured by energy-dispersive X-ray spectrometry (accessory to SEM). The hardness along the depth of the transverse section was measured using an HX-1000-type micro-Vickers hardness tester with a load of 50 g and loading time of 10 s. A rhombic diamond indenter was used in the experiments, where three indentations were induced using each load. Other parameters were based on those specified in the standard ASTM E384-08. Wear resistances of the Co-based alloy/Ni/n-Al_2_O_3_ duplex coatings and Cu substrates were evaluated using a pin-on-disk apparatus (MMS-1G). The dimensions of the pin were Ф 6 mm × 12 mm, and a slide disk (GCr15 steel, hardness of HRC 60, Peking, China) with a diameter of 70 mm was used as the counter body. Wear tests were performed at room temperature, at a sliding speed of 2 m/s, at a constant normal load of 100 N, and under dry sliding conditions. Each test was conducted for 20, 30, 40, and 60 min with sliding distances of 1.457, 2185, 2914, and 4371 km, respectively. Sample surfaces were ground and polished with 2500-grit paper. In accordance with the standard ASTM G99-95a, the volume loss was measured from a change in the pin height during the wear tests and more than six replicate wear experiments were conducted. Surfaces of the wear specimens were observed by SEM.

## 3. Results and Discussion 

### 3.1. Morphology and Microsturcture of Coatings

**Figure 2:**
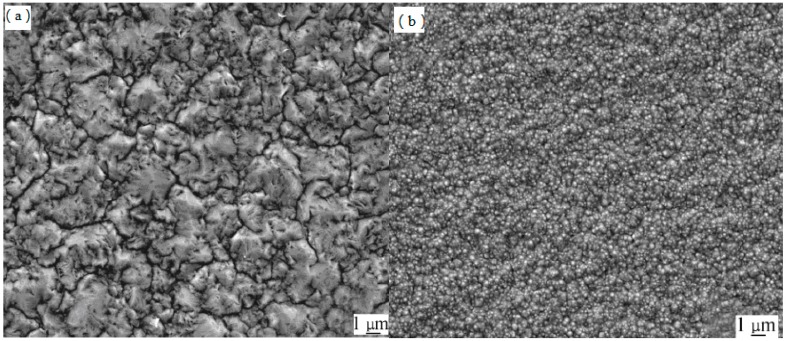
SEM micrographs of electroplating layers: (**a**) Ni layer and (**b**) Ni/n–Al_2_O_3_ layer.

Surface micrographs of electrodeposited coatings prepared by dc methods are shown in Figure 2. Nanometer-scale Al_2_O_3_ particles on Ni grain boundaries restricted the growth of Ni grains during electrodeposition, which refined Ni grains. The surfaces of Ni/Al_2_O_3_ composite coatings were therefore smoother and the composite coatings contained finer, more uniform, denser Ni crystals compared with pure Ni coatings, as shown in Figure 2. These results indicate that the Ni/n-Al_2_O_3_ coatings are more suitable as interlayers compared with pure Ni coatings. 

**Figure 3:**
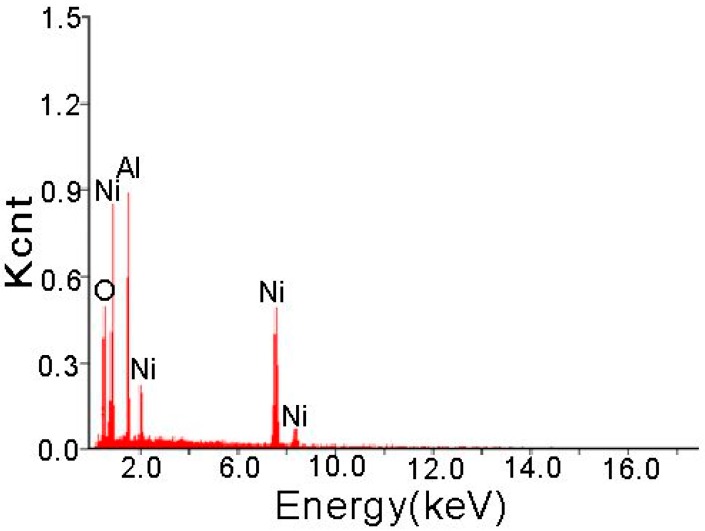
EDX spectrum of the Ni/n–Al_2_O_3_ layer.

**Figure 4:**
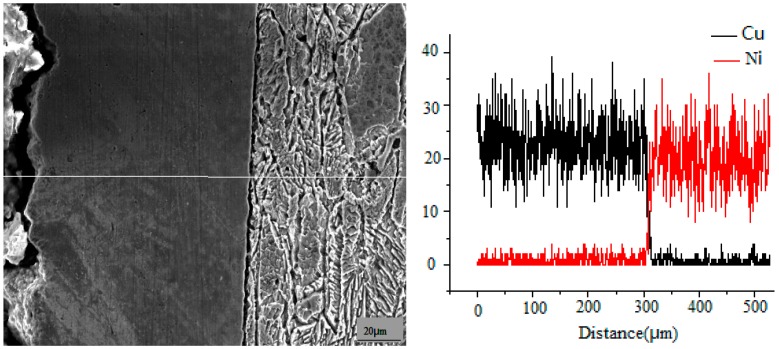
Distributions of Cu and Ni along the cross-section of the Ni/n–Al_2_O_3_ layer.

Figure 3 shows the EDX spectrum of a composite coating with 30 g/L Al_2_O_3_ added. This spectrum shows that a certain amount of Al_2_O_3_ nanoparticles was dispersed in electroplating layers. The cross-sectional elemental distributions of Cu and Ni from the Cu substrates to Ni/n–Al_2_O_3_ coatings are demonstrated in Figure 4. Cu and Ni are observed only in the substrates and coating, respectively, and the substrates and coating are mechanically bonded at their interface. 

**Figure 5:**
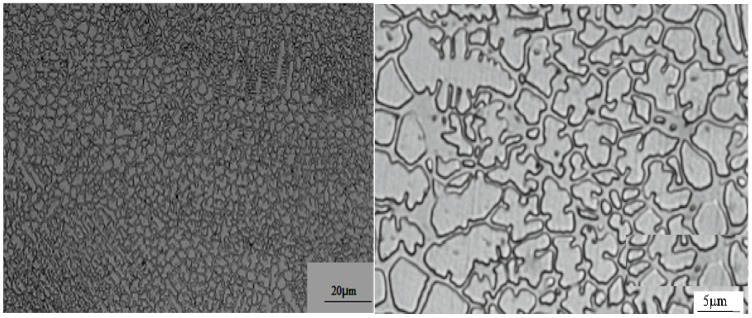
Surface micrographs of the Co-based laser cladding coating.

Surface micrographs of a Co-based laser cladding coating are shown in Figure 5. They reveal that this coating is smooth and defect free and that fine equiaxed grains are the predominant microstructure of the coating.

**Figure 6:**
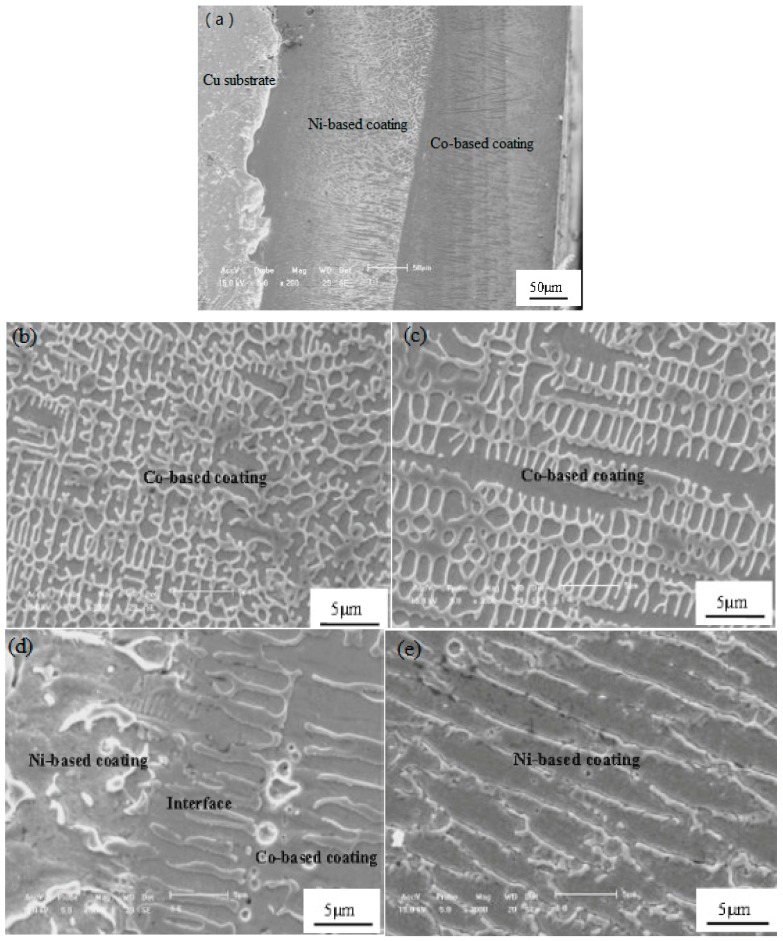
Cross-sectional micrographs of the Ni–Co duplex coating: (**a**) microstructure of duplex coating; (**b**) surface microstructure of Co-based coating; (**c**) middle microstructure of Co-based coating; (**d**) bottom microstructure of Co-based coating and (**e**) Ni/Al_2_O_3_ intermediate coating.

Figure 6 displays cross-sectional micrographs of the Co-based alloy–Ni/n–Al_2_O_3_ duplex coating after laser cladding. As evident in Figure 6a, the coating consists of a Co-based alloy layer and Ni/n–Al_2_O_3_ interlayer with thicknesses of approximately 250 μm and 200 μm, respectively. Fine dendrite crystals are observed in the Co-based laser cladding layer with no evident defects. A columnar crystal structure is observed near the boundary of the Co-based cladding coating and Ni/n–Al_2_O_3_ coating; on the other hand, a compact and disorientated structure is observed at the top of the Co-based coating surface. The magnified micrographs of the Co-based cladding layer and interlayer are shown in Figure 6b–e.

**Figure 7:**
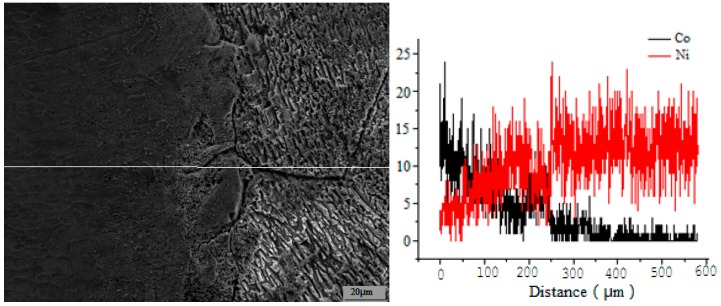
Distribution of Co and Ni along the cross-section of the Ni–Co duplex coating.

The grain morphologies of the Ni/Al_2_O_3_ layer and Co-based cladding layer were governed by their consolidation conditions. During rapid remelting and recrystallization LSC, heat always transfers from the surface of the duplex coating to bulk cold substrate and dendrites solidify parallel with the direction of this thermal transmission, resulting in dendritic growth of the Ni/Al_2_O_3_ layer and Co-based cladding layer. The upper region of the laser cladding layer experiences rapid cooling, resulting in rapid crystallization and a fine-grained texture. In the middle region of the laser cladding layer, heat transfer to the substrate is difficult because of the obstacle of the bottom region; isometric dendrite structures are consequently formed in this region. The elemental distributions of Ni and Co along the transverse section of the Ni–Co duplex coating are demonstrated in Figure 7.

**Figure 8:**
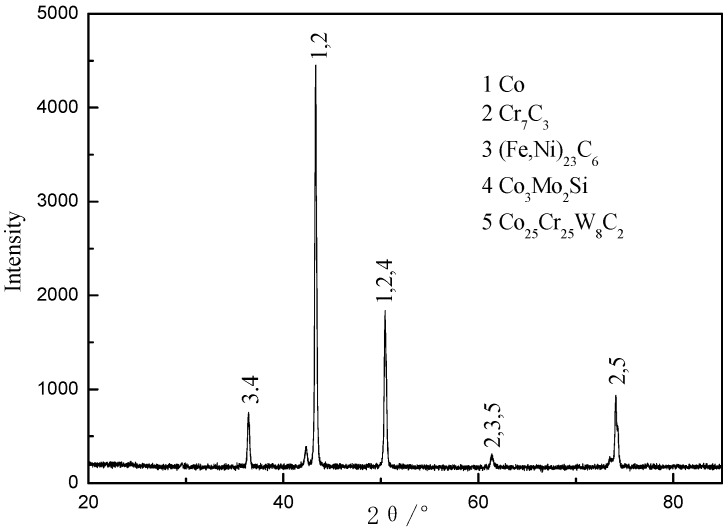
X-ray diffraction (XRD) pattern of the Co-based cladding layer.

The results show that the mutual diffusion of elements occurs between the laser cladding layer and interlayer and that the interface strength is a consequence of metallurgical bonding between these layers. The X-ray diffraction (XRD) pattern in Figure 8 indicates that a Co-based sosoloid is the main constituent of the Co-based cladding layer and is accompanied by (Fe,Ni)_23_C_6_, Cr_7_C_3_, Co_3_Mo_2_Si, and Co_25_Cr_25_W_8_C_2_ hard phases. These results demonstrate that the Ni–Co duplex coatings are reinforced by (Fe, Ni)_23_C_6_, Cr_7_C_3_, and other hard phases and exhibit no obvious flaws, porosity, or other defects.

### 3.2. Microhardness and Wear Resistance of the Coatings

**Figure 9:**
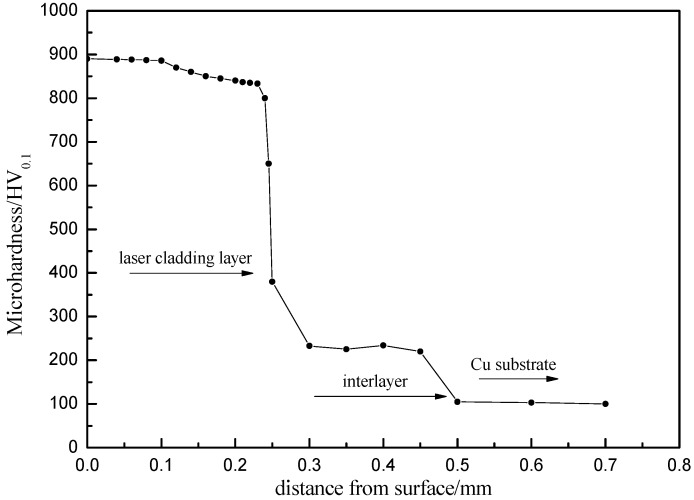
Variation of the microhardness profile along the cross-section of the Ni–Co duplex coating.

Figure 9 shows a variation of the microhardness profile along a cross-section of the Ni–Co duplex coatings. Clearly, the microhardness increases from the Cu substrate to interlayer and laser cladding layer. The hardness of the coatings increases with decreasing distance from the surface. Therefore, the hardness reaches its maximum at the coating surface, where a hardness value of approximately 886 HV was measured. An average hardness of 845.6 HV was obtained for a surface coating more than 250-μm thick and corresponding to the Co-based cladding layer; this hardness is 8.2 times greater than that of the Cu substrate (102.6 HV) and 3.7 times greater than that of the Ni/n–Al_2_O_3_ electroplated layer (228 HV). Compared with the substrate and interlayer, the hardness of the Co-based cladding coating was significantly improved because of the formation of a Co-based solid solution, Cr_7_C_3_, (Fe,Ni)_23_C_6_, and other strengthening phases. In addition, the Ni–Co duplex coating exhibited a relatively uniform distribution of hardness, which indicates the absence of sharp demarcation in material properties across the interface. That is, an electroplated Ni/Al_2_O_3_ interlayer between the Co-based cladding coating and Cu-based matrix is an excellent transitional zone.

**Figure 10:**
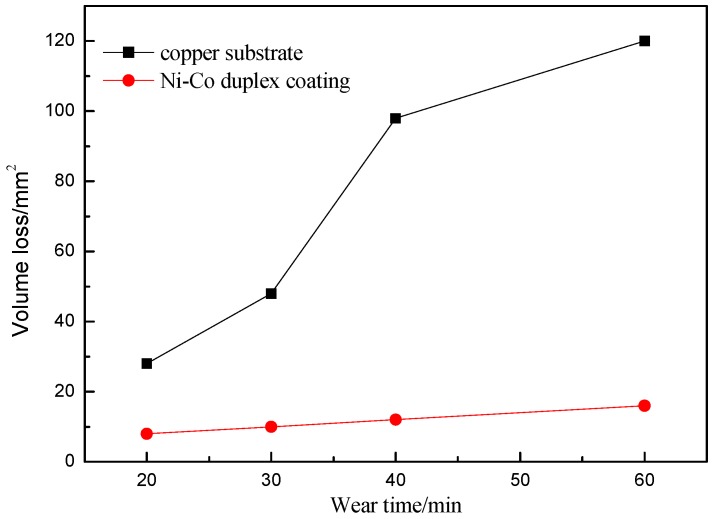
Wear resistance of the Ni–Co duplex coating.

**Figure 11:**
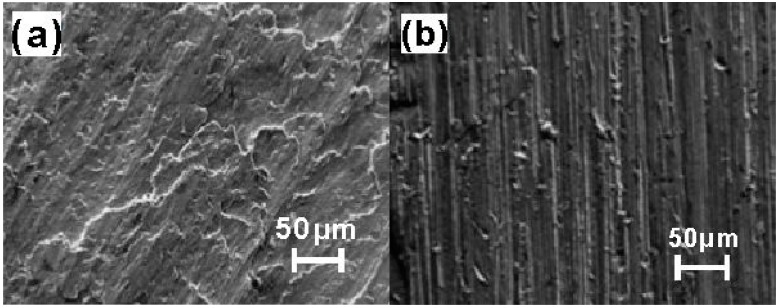
Worn surface morphologies of (**a**) the Cu substrate and (**b**) the Ni–Co duplex coating.

The wear resistance of the Ni–Co duplex coating is shown in Figure 10. The volume loss of the Ni–Co composite coating is clearly lower than that of the Cu-based substrate. The volume loss of the Cu-based substrate is 3.5 times greater than that of the Ni–Co composite layer after a 20-min wear test, and this value increases to 7.5 at 60 min. These results clearly demonstrate that the wear resistance of the Cu-based substrate can be substantially modified by laser cladding Ni–Co compact coatings, mainly because of the high hardness, strong bonding, and crack-free microstructure of such coatings. Therefore, the Ni–Co duplex coatings can improve the hardness and wear resistance with no obvious increase in the friction coefficient of the coatings. Figure 11 shows the worn surface morphology of a Cu-based substrate and Ni–Co composite coating after a 60-min wear test. During the wear process, the Cu substrate surface became rough with numerous adhesive patches; plastic deformation obviously occurred. Plate-like debris was pulled away from the Cu surface, which illustrates that the Cu substrate suffered severe adhesive wear. In contrast, the worn surface of the Ni–Co duplex coating was relatively smooth with only mild scratches and few adhesive wear characteristics; these observations indicate improved adhesive wear, consistent with the results in Figure 9. Plastic deformation is difficult in the duplex coatings during the dry sliding wear process because of their relatively high hardness. Therefore, their excellent resistance to plastic deformation and scraping results in improved resistance to plastic erasing and adhesive wear. 

## 4. Conclusions

Defect-free Ni–Co duplex coatings were successfully fabricated on Cu substrates via successive laser-cladded Ni/n–Al_2_O_3_ and Co-based coatings using a 6-kW transverse-flow CO_2_ laser. The Co-based coating and copper substrate were bonded by electroplating an Ni/n-Al_2_O_3_ intermediate. The Ni–Co duplex coatings exhibited high hardness with no obvious defects. At the top of Ni–Co composite coating surfaces, (Fe,Ni)_23_C_6_, Cr_7_C_3_, and other hard phases reinforced Co-based dendritic crystals and compact equiaxed grains were formed, contributing to an average coating hardness of 845.6 HV; this hardness was approximately 8.2 times greater than that of the Cu-based substrate (102.6 HV). During sliding wear tests, the volume loss of the Cu substrates was 3.5 times greater than that of the Ni–Co composite coatings at 20 min, and this value increased to approximately 7.5 at 60 min. The Ni–Co duplex coatings exhibited greater wear resistance and lower friction coefficient compared with the Cu substrates.

## Figures and Tables

**Table 1:** Composition of the plating solution.

Component	NiSO_4_·6H_2_O	Al_2_O_3_	NiCl_2_·6H_2_O	CH_3_(CH_2_)_11_SO_3_Na	H_2_BO_3_
g/L	280	30	45	0.1	40

**Table 2:** Composition of the Co-based alloy powder.

Element	Co	Ni	Cr	Si	W	Fe	C
Wt %	50	12	25	3	8	2	0.75
